# Entrepreneurship in family firms: an updated bibliometric overview

**DOI:** 10.1007/s11846-023-00650-z

**Published:** 2023-03-22

**Authors:** Muhammad Anwar, Thomas Clauss, Natanya Meyer

**Affiliations:** 1 Witten Institute for Family Business, Witten/Herdecke University, Witten, Germany; 2 University of Johannesburg, Johannesburg, South Africa; 3 Department of Innovation and Technology, University of Southern Denmark, Odense, Denmark

**Keywords:** Bibliometric coupling, Bibliographic coupling, Co-citation analysis, Corporate entrepreneurship, Entrepreneurial orientation, Entrepreneurship, Family business, Family firm, L26, L25, Z11

## Abstract

Research on entrepreneurship in family firms has grown exponentially over the past two decades. Due to the various theoretical perspectives and contexts found here however, this body of research remains fragmented, with a unified understanding of the current state of knowledge and the opportunities for future research in the field continuing to lack. In this study, we address this gap by conducting an updated bibliometric analysis of the research on entrepreneurship in family firms. Here we integrate two different bibliometric methods to provide a more comprehensive picture of the field, unveiling its intellectual foundations and current research discourses and how these two are related. To do this, we first conduct a co-citation analysis clustering the intellectual foundations of the research on entrepreneurship in family firms. Second, a bibliographic coupling of recent publications from 2010 to 2021 provides a transparent structure of current research discourses. Third, analyzing which intellectual foundations are primarily cited in each current research stream unveils the dominant theoretical paradigms in the current state of research. Analyzing 570 published studies, we identified four intellectual foundations of entrepreneurship in family firms: socioemotional wealth (SEW), entrepreneurial orientation, family-embedded resources, and agency theory. The current research can be clustered into seven main discourses: entrepreneurial motivation, gender and success, entrepreneurial orientation, individual and firm-level characteristics, the family embedded network, family firm internationalization, and family heterogeneity. An integrative network diagram provides an overview of the research field’s development while also identifying the gaps to be addressed by future research.

**Supplementary Information:**

The online version contains supplementary material available at 10.1007/s11846-023-00650-z.

## Introduction

Family firms are one of the most prevalent and oldest business forms (Kayid et al. 2022), accounting for more than 80% of worldwide businesses (Gagne et al. 2021). Although basically every firm will have started as an entrepreneurial venture when founded, the questions of whether and why family firms remain entrepreneurial is an ongoing debate in the literature. Recently, Minola et al. (2021) emphasized that the research interest in entrepreneurial family firms has grown consistently since Kellermanns and Eddleston’s (2006) seminal work on the determinants of corporate entrepreneurship in family firms. Research has identified several factors influencing entrepreneurial activities within the family firm domain. For instance, Bettinelli et al. (2017) demonstrate that contextual level, family level, firm level and individual level drivers influence entrepreneurship in family firms.

Various terms are used to describe entrepreneurship in existing firms. (G. Lumpkin & G. Dess, 1996a) distinguished between entrepreneurship and entrepreneurial orientation by referring to *entrepreneurship* as “new entry” (entering new or established markets with new or existing goods or services) and *entrepreneurial orientation* as the practices, processes, and decision-making activities that lead to new entry. Generally, entrepreneurial orientation is considered as the intentions and actions of key players functioning in a vibrant reproductive process aimed at new venture creation (G.T. Lumpkin & G.G. Dess, 1996b). Jennings and Lumpkin (1989) defined *corporate entrepreneurship* as “the extent to which new products and/or new markets are developed. An organization is entrepreneurial if it develops a higher than average number of new products and/or new markets” (p. 489).

While in fact two major aspects of research on family firms’ entrepreneurship have been contested, their insights remained fragmented. For instance, one research stream claims that family firms are more entrepreneurially-oriented and engaged in innovative products, processes, and services (e.g.,Kansikas et al., 2012; Radu-Lefebvre et al., 2022; Zahra, 2005; Zahra et al., 2004), a result of their high level of family engagement (Zahra 2003), kinship, and social ties (Barney et al. 2003; Weimann et al. 2021). On the other hand, researchers have described how family firms lack entrepreneurial behavior (Autio and Mustakallio 2002; Bertrand and Schoar 2006; Block et al. 2013). The reasoning behind this could be that family firms invest less money in innovative activities than non-family firms (Gomez-Mejia et al. 2010; Schulze et al. 2003). Other reasons also include the process of passing control from founding to later generations (Block et al. 2013; Le Breton-Miller and Miller 2011), family control (Carney 2005), wealth preservation (Carney 2005; Chrisman et al. 2005), and the continuation of generational and family leaders’ tenure (Kellermanns et al. 2008). Considering the inconsistencies among the attitudes and decisions of family members towards entrepreneurship, we see significant value in providing an updated overview of the existing research at the junction of family firms’ entrepreneurship (Raitis et al. 2021). The contrasting visions of how family members are engaged in entrepreneurial activities lead to substantial heterogeneity in family businesses (Chrisman et al. 2012; Jennings et al. 2013). As a result, an incoherent and complex stream of empirical studies on the causes and benefits of family firms’ entrepreneurship has been put forward (Bağış et al. 2022; Bettinelli et al. 2017; Cardella et al. 2020; Kraus et al. 2012; Lumpkin et al. 2011; Wang et al. 2022). A few studies have mapped the research on family firms’ innovation, such as Baltazar et al. (2023) who systematically reviewed 32 articles and identified three domains involving the success and innovation processes in family firms: (a) the impact of succession on innovation; (b) succession and sharing of knowledge; and (c) obstacles to innovation. Using a bibliometric approach, Wu et al. (2022) focused on the rapid phase of family firms in globalization and studied innovative measures in family firms during globalization. Innovation is a single dimension of entrepreneurship, and it does not necessarily encompass the entire issue of entrepreneurship in family businesses. According to Schmitz et al. (2017), innovation is more aligned to novelty creation at the beginning of a process, while entrepreneurship is more associated with value creation at the end of one. To be sure, there are coherently overlapping elements of both of these ideas, even though their differences are also clear. This is why research in family firms’ entrepreneurship needs thorough investigation to recognize the most- and least-researched areas in the field.

The existing literature still lacks a clear direction for theorizing its research. Despite an increased number of studies so far, to our knowledge, none to date have been carried out to describe the intellectual foundations and current streams of research in family firm entrepreneurship. This constitutes a clear research gap, as a better understanding of the structure of the literature on entrepreneurship in family firms, together with an exploration of the underlying theoretical foundations, will facilitate a more comprehensive theoretical discourse and unveil areas that require future research attention. López-Fernández et al. (2016) conducted a bibliometric analysis of this literature from 1992 to 2011, and because of the rapidly growing number of publications in the field, updating this analysis to include the research of the past 10 years was deemed necessary. Doing this can significantly contribute to a better understanding of family firm entrepreneurship. We deem this particularly important based on the observation of (Aldrich et al. 2021) that the number of studies on entrepreneurship in family firms has steadily increased in recent years, and that new methodological (e.g. Giner & Ruiz, 2022; Riar et al., 2022) and theoretical (e.g. Minola et al., 2021; Zahra, 2022) approaches have entered the field.

To close this gap, this study conducts a multi-step bibliometric analysis on entrepreneurship in family firms. First, we identify the intellectual foundations of family firms’ entrepreneurship through co-citation analysis. Second, we identify the most prevalent current research streams via an additional bibliographic coupling analysis. Third, we link these two analyzes to determine which theoretical foundations the current research activities are based on, and which “white” spaces exist in this research landscape.

This research contributes to the field by focusing on three points. We first achieve a more unified understanding of family firms’ entrepreneurship by reviewing the recent literature from 2010 to 2021 (found in the WOS and Scopus), identifying the most emergent and contemporary trends. The bibliographic coupling analysis reveals the current state and research trends in family firm entrepreneurship, and can assist researchers in systemizing and better understanding how this field might be developing. The insights into the theoretical foundations based on co-citation analysis facilitate scholars in understanding the progressive work in the field. Second, we created a network diagram to provide an integrated overview of the field’s development, which enabled us to identify the missing links to be addressed by future research. The insights of this research can be very useful for emerging researchers and scholars entering the field, and for existing researchers as they advance their understanding of family firm entrepreneurship. Our work will hopefully allow future scholars to understand the missing areas and white spaces in the field of family firms’ entrepreneurship in various industries.

## Entrepreneurship and family firms: a brief theoretical overview

Family firms and entrepreneurship fields consist of two separate and unique, albeit overlapping domains (Hoy and Verser 1994). When the two domains of “family” and “entrepreneurship” intersect, a potential conflict (family values and emotional attachment to family assets vs. the entrepreneurial process) arises. Unless precautionary measures are taken to separate family from business, this conflict may affect the entrepreneurial process in family firms (Craig and Lindsay 2002). The family influence characterizes family firms, and is manifested through family ownership in many cases in combination with family members as part of fundamental governance and management tasks (Astrachan et al. 2002). Family firm entrepreneurship (Bettinelli et al. 2017 p.506) is defined as “the firm-level entrepreneurial attitudes and activities that occur when a family is considerably involved in an established organization.”

Family businesses are considered an important engine of entrepreneurship across the globe (Eze et al. 2021). Entrepreneurial activities and education are vital for family businesses to expedite global economic growth (Soares et al. 2021). The discussion on family firm entrepreneurship is manifested in terms of corporate entrepreneurship and entrepreneurial orientation (Bettinelli et al. 2017; Cruz and Nordqvist 2012; Minola et al. 2021; Randolph et al. 2017; Zahra et al. 2004). Scholars have furthermore discussed entrepreneurial activities in family businesses regarding different aspects. For instance, Bettinelli et al. (2017) presented individuals, contextual, and firm-level factors in a framework that highlights the backgrounds, consequences, and procedures of family firms’ entrepreneurship. Cruz and Nordqvist (2012) revealed that second-generation-controlled family firms have a high level of entrepreneurial orientation where highly competitive environments exist, while third- and later-generation family firms pursue entrepreneurial activities when non-family management teams are involved.

Focusing on some of the most influential studies in the field, Kellermanns and Eddleston (2006) indicate that a willingness to change and technological opportunity favorably influence corporate entrepreneurship in family businesses, while strategic planning is a significant moderator of generation involvement and technological opportunity regarding family firms’ entrepreneurship. Webb et al. (2010) demonstrate that family involvement in terms of identity, nepotism, justice, and conflict creates differences in the strategic entrepreneurship between family-controlled and non-family firms. Eddleston et al. (2012) studied the differences in family firms’ entrepreneurial behaviors caused by strategic decision-making, long-term orientation, participative governance, and human capital. Jaskiewicz et al. (2015) revealed that entrepreneurial legacy motivates transgenerational entrepreneurship in family firms. Another study by Zellweger and Sieger (2012a) claims that the existing questions about *autonomy*, *innovativeness*, *risk-taking*, *proactiveness*, and *competitive aggressiveness* are not satisfactory, and require further development in the field of family businesses. Naldi et al. (2007) differentiated between family and non-family firms based on the interaction of risk-taking, proactiveness, and innovativeness. Similarly, considering recent studies in family firms’ entrepreneurship, Minola et al. (2021) advance the theoretical ground for corporate entrepreneurship in family firms by employing different domains such as ontology, epiphany, and heterogeneity. Calabrò et al. (2022) concluded that familiness human, social, and financial capital resources, along with the degree of entrepreneurial orientation significantly influence transgenerational entrepreneurship and family business performance. Moreno-Menéndez et al. (2022) checked entrepreneurial activities pre- and post-COVID, revealing that entrepreneurship has seen a boost following COVID, even though economic decline and organizational change significantly affect entrepreneurial actions in family business. Gjergji et al. (2022) connected the dimensions of socioemotional wealth to entrepreneurial behaviors in family firms, showing that except for “emotional attachment among family members,” other dimensions play a positive role. Strobl et al. (2022) described how family-owned SMEs benefit from entrepreneurial orientation under entrepreneurship leadership, while environmental dynamism can impede their entrepreneurship. Chen et al. (2022) found that family control negatively influences entrepreneurial activities in family firms. Seyed Kalali (2022) indicated that long-term orientation positively influences innovativeness and proactiveness while negatively influencing risk-taking activities in family firms. All of these works serve to show that research on family firms’ entrepreneurship takes a multitude of factors into account.

In particular, entrepreneurship in family firms is studied according to three concepts: innovation, strategic renewal, and corporate entrepreneurship or venturing (Riar et al. 2022). Contextual (e.g. Discua Cruz et al., 2021), firm level (e.g. Moreno-Menendez et al., 2022), family level (e.g. Hernández-Perlines et al., 2019), and individual level (e.g. Boling et al., 2016) drivers influence the entrepreneurship and outcomes of family businesses (Bettinelli et al. 2017; Iturrioz-Landart et al. 2022; Minola et al. 2021; Radu-Lefebvre et al. 2022; Wang et al. 2022).

Generally, corporate entrepreneurship is particularly important in family businesses because it not only contributes to short-term value creation, but is an investment in future generations (Cruz and Nordqvist 2012) as it fosters transgenerational entrepreneurship (Jaskiewicz et al. 2015; Marchisio et al. 2010). More recently, however, new challenges and opportunities have accelerated the importance of entrepreneurship in family firms. The COVID-19 pandemic has pushed family businesses to exploit entrepreneurial opportunities in international markets (Zahra 2022). Research has shown that, during crises, family businesses emphasize business model innovation (Kraus, Clauss, et al., 2020) and strategic renewal (Issah et al. 2023). More specifically, studies have recently addressed social entrepreneurship (e.g. Khan et al., 2022) and digital entrepreneurship activities that benefit family businesses (Upadhyay et al. 2022). It was furthermore shown that entrepreneurship in family business is not limited to the top management level, but that individuals and family members across many (if not all) levels are incentivized to engage in new venture creation and startup activities (Riar et al. 2022). To summarize, entrepreneurship in family firms today is influenced by a multitude of new factors (Aldrich et al. 2021; Zahra 2022), leading to a substantially enriched and more complex debate.

We understand that several factors have been introduced in the existing literature that foster or hinder entrepreneurial activities in family firms. However, as discussed earlier, this growing literature has often ignited the question regarding to what extent family firms are engaged in entrepreneurial activities. Although our study does not specifically aim to answer this question, it does strive to identify its intellectual foundations and highlight the emerging research streams to date, focusing on important aspects leading to family firm success. These include family firm entrepreneurship, entrepreneurial orientation, entrepreneurial activities, and corporate entrepreneurship. In the process of accomplishing this, we identify possible “white spaces” that can lead to future research.

## Methodology

This study encompassed a number of steps, including defining the research design, bibliometric methods, software, database, search term, data limitations, and data screening.

### Research design

This study focused on a bibliometric approach. “Bibliometrics is a quantitative analysis of gross bibliographic units such as books, journals, articles, and the like” (Donohue 1972 p.313). The number of systematic and bibliographic studies has been increasing in the domains of entrepreneurship, innovation, and strategic management thanks to their potential to provide systematic and structured overviews of past research trends and opportunities for future research (e.g., Kraus, Breier, et al., 2020; Kraus et al., 2022; Lampe et al., 2020). In the present bibliographic study, we determine the theoretical foundation and the most relevant literature in the field of family firm entrepreneurship. Maseda et al. (2022) consider co-citation and coupling analyzes as the most relevant techniques in bibliometric studies because they enable scholars to understand the past and present debate in a particular field. In the field of family business, numerous scholars have employed the same technique in different research areas. For instance, Baltazar et al. (2023) employed VOSviewer to explore current streams of research in the fields of innovation, succession, and family businesses. Wu et al. (2022) conducted a co-citations and coupling analysis through VOSviewer in the field of family business and digitalization. Casado-Belmonte et al. (2021) mapped family firms’ innovation through co-citations, and co-wording analysis using VOSviewer. Hence, our analysis combined two approaches to outline the publications in the fields of co-citation and bibliographic coupling analysis:

1. *Co-citation*: This is when two articles are cited independently by one or more articles (Culnan 1986). In other words, co-citations record the number of articles that have cited a particular pair of papers, which is deemed as a measure of the similarity of their content.
2. *Bibliographic coupling*: This is when two articles cite a certain number of common articles in their bibliographies. This number of shared references within two papers indicates the degree to which these two articles are similar regarding their subject matter (Vogel and Güttel 2013).

The main differences between these two methods are that, first, as co-citations are established through the citations of every newly published work, they are not static and can change over time (Eto 2013). On the other hand, bibliographic couplings between two articles always remain stable (Caputo et al. 2019). Second, co-citation analysis analyzes cited articles, naturally examining older articles that create the foundations for newer work. A new publication not yet cited cannot be part of a co-citation analysis (e.g. Loi et al., 2016). This requirement however does not exist for bibliographic couplings that are established for every publication. So bibliographic coupling analysis in particular facilitates the analysis of recent research streams (Belussi et al. 2019). Indeed, these two methods produce different outcomes, and they are a supplement to each other rather than a substitute (Jarneving 2005). Several bibliometric studies have successfully applied these methods as complementary (Casprini et al. 2020; Ferreira 2018; Vogel and Güttel 2013).

We used the software VOSviewer for our analysis. This software was considered a good selection compared to other available software packages, and used in most of the latest bibliographic studies within the same field (e.g., Belussi et al., 2019; Casprini et al., 2020; Ferreira, 2018). Although analytically, this software does not significantly differ from other available tools, VOSviewer does in fact unfold its strength in visualizing relationships between co-cited and coupled research articles (Donthu et al. 2021). We followed the method of Echchakoui (2020), who explained the merging process in detail. VOSviewer enables the analysis and visualization of co-authorship, citations, co-occurrences, bibliographic couplings, and co-citations. It displays all topics with unique colors in the figures, and provides information about the highest co-cited and coupled documents over time. It displays bibliometric maps as a multidimensional scaling that provides detailed information about research insights.

### Database and search protocol

We used the Scopus and WOS databases to identify the literature for our bibliometric analysis for the years 2010–2021. Scopus is considered the largest database of international publishers and scientific peer-reviewed literature (Luo et al. 2020). WOS contains the highest quality peer-reviewed journals worldwide (Kullenberg and Kasperowski 2016), and is also considered the most reliable database for bibliographic studies because it does not exhibit bias toward publishers (Falagas et al. 2008). Both databases provide comprehensive literature on entrepreneurship and business topics, and are mostly used for bibliometric searching (Gupta et al. 2021; Lampe et al. 2020).

Identifying an appropriate search term is crucial for comprehensively capturing the relevant literature and avoiding polluted samples (Ye et al. 2020). As our study’s focus is on entrepreneurship and family firms, we used the following specific search terms: (“entrepreneur*”) AND (“Family firm*” or “Family Business*” or “Family Enterprise*” or “Family Organization*” or “Family Organisation*” or “Family Control*” or “Family own*”) with a selection of titles, abstracts, and keywords. As the broad term “entrepreneur” generally encapsulates all entrepreneurially-related aspects such as “entrepreneurship”, “corporate entrepreneurship”, and “entrepreneurial orientation”, we deemed this single search term sufficient. We further specified the family firm aspect by capturing the various terms used for family firms and/or family-owned organizations adapted from previous studies (Alayo et al. 2021; López-Fernández et al. 2016). After the initial search, we manually screened the data to attenuate the threat of contaminated literature (e.g., papers that have studied only family entrepreneurs/enterprises instead of entrepreneurship or entrepreneurial activities in family firms) that were generated by the search term “entrepreneur*”.

To concentrate on the most useful research in the study, we filtered our search to include only regular articles, excluding editorials, commentaries, and book chapters. Similarly, we restricted our search to only academic peer-reviewed journals, while other articles from conferences, commercial magazines, book series, and trade journals were eliminated due to their unclear scientific contributions (Ye et al. 2020). Articles written in English with the period data from 2010 to 2021 were included.

Our initial search yielded 770 documents from Scopus and 696 from the WOS database dating 2010–2021. After checking for duplication, 326 documents indexed in both Scopus and WOS were excluded, resulting in 1,140 documents. We then manually screened their titles, abstracts, and keywords to check if there were articles that did not directly relate to the field of family firm entrepreneurship; 570 articles that did not focus on a topic related to entrepreneurship in family firms were discarded. These articles were initially identified as mentioning the words entrepreneur, entrepreneurship, or entrepreneurial intention in the abstract or keywords, but did not address the main topic of entrepreneurship in family businesses. If there was doubt whether the paper addresses family firm entrepreneurship, we read the entire paper to determine if it related to entrepreneurship in family firms. Following this manual screening, we retained 570 articles (50% of 1,140) for the analysis.

## Analyzes and results

### Descriptive analysis of the field

**Fig. 1:**
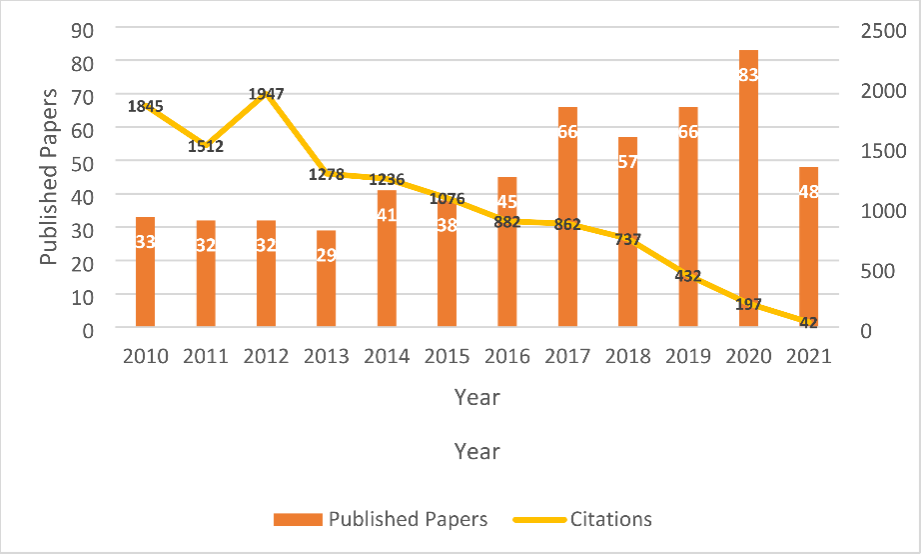
Total and screened documents and citations

Table 1 shows the number of papers (retained from Scopus and WOS) from the years 2010–2021. The number of papers published over the years increased while citations decreased (except for one or two years). The trend of publications and citations is given in Fig. 1.

**Table 1:** Total and screened documents and citations

Year	Screened Papers	Screened Citations
2010	33	1845
2011	32	1512
2012	32	1947
2013	29	1278
2014	41	1236
2015	38	1076
2016	45	882
2017	66	862
2018	57	737
2019	66	432
2020	83	197
2021	48	42
Total	570	12,046

Table 2 shows the 20 top-cited papers in the field of family firm entrepreneurship. The research by Zellweger et al. (2011) emphasizes the basic mode of entrepreneurship: starting new careers or staying in existing ones. The second most cited paper by Lumpkin et al. (2010) sheds light on the long and short-term role of entrepreneurial orientation on family firm performance. Moreover, Cruz and Nordqvist (2012) scrutinized how generations influence the relationship between internal and external factors and entrepreneurial orientation.

**Table 2:** Top 20 cited papers

No	Documents*	Citations	No	Documents*	Citations
1.	Zellweger et al. (2011), *JBV*	253	11.	Chirico et al. (2011), *SEJ*	171
2.	Lumpkin et al. (2010), E&*RD*	252	12.	Miller and Le Breton–Miller (2011), *ETP*	162
3.	Cruz and Nordqvist (2012), *SBE*	233	13.	Casillas et al. (2010), *FBR*	157
4.	Stewart and Hitt (2012), *FBR*	232	14.	Sciascia et al. (2012), *SBE*	145
5.	Zellweger et al. (2012), *FBR*	229	15.	Chirico and Nordqvist (2010), *ISBJ*	139
6.	Shepherd et al. (2015), *JOM*	218	16.	Chlosta et al. (2012), *SBE*	134
7.	Ucbasaran et al. (2013), *JOM*	214	17.	Discua Cruz et al. (2013), *ETP*	133
8.	Jaskiewicz et al. (2015), *JBV*	212	18.	Casillas and Moreno (2010), *E&RD*	131
9.	Nordqvist and Melin (2010), *E&RD*	198	19.	Eddleston et al. (2012), *ETP*	129
10.	Zellweger and Sieger (2012b), *SBE*	190	20.	Salvato et al. (2010), E&*RD*	122

*JBV = Journal of Business Venturing; E&RD = Entrepreneurship & Regional Development; SBE = Small Business Economics; FBR = Family Business Review; JOM = Journal of Management; SEJ = Strategic Entrepreneurship Journal; ETP = Entrepreneurship Theory and Practice; ISBJ = International Small Business Journal.

Table 3 illustrates the most productive journals and authors. Concerning the number of publications in journals, it can be seen that the *Journal of Family Business Management* is the most productive, followed by *Entrepreneurship Theory and Practice*, which published 28 and 27 documents, respectively. A gradual decline is observed in the number of documents in the rest of the journals in the table. Regarding citations, *Family Business Review*, *Small Business Economics*, *Entrepreneurship Theory and Practice*, and *Entrepreneurship & Regional Development* are the leading journals on the list. Considering the most productive author in terms of published papers, Nordqvist and Kellermanns have published 10 papers, Zellweger has published nine, with this number gradually declining for the other authors.

**Table 3:** The most productive journals and authors

No	Journal	Documents	Citations	Authors	Papers	Citations	Average Citations Per Article
1.	Journal of Family Business Management	28	113	Nordqvist M	10	900	100
2.	Entrepreneurship Theory and Practice	27	1209	Kellermanns FW	10	348	34.80
3.	Journal of Family Business Strategy	25	722	Zellweger T	9	1128	125.33
4.	International Entrepreneurship and Management Journal	24	218	Eddleston KA	8	331	41.375
5.	Journal of Small Business Management	22	451	Chirico F	8	712	89.00
6.	Family Business Review	21	1312	Peters M	8	170	21.25
7.	Small Business Economics	21	1240	Minola T	7	86	12.29
8.	International Journal of Entrepreneurial Behaviour and Research	21	355	De Massis A	7	292	41.71
9.	Entrepreneurship & Regional Development	18	1009	Kallmuenzer A	7	146	20.86
10.	International Journal of Entrepreneurship and Small Business	16	177	Kraus S	7	251	35.86
11.	Journal of Business Research	15	215	Kammerlander N	7	101	14.43
12.	Business History	12	123	Hamilton E	7	359	51.29
13.	Strategic Entrepreneurship Journal	9	372	Campopiano G	6	102	17.00
14.	Management Decision	8	73	Llanos-Contreras O	6	49	8.17
15.	Journal of Management Studies	7	140	Lumpkin GT	6	386	64.33
16.	Journal of Business Venturing	7	699	Sieger P	6	616	102.67
17.	International Small Business Journal-Researching Entrepreneurship	7	357	Clinton E	6	111	18.50
18.	Journal of Small Business and Entrepreneurship	7	57	Holt DT	6	142	23.67
19.	International Journal of Entrepreneurship and Innovation Management	7	105	Ratten V	6	109	18.17
20.	International Journal of Gender and Entrepreneurship	7	102	Iturralde	5	108	21.80

### Co-citations analysis

**Fig. 2:**
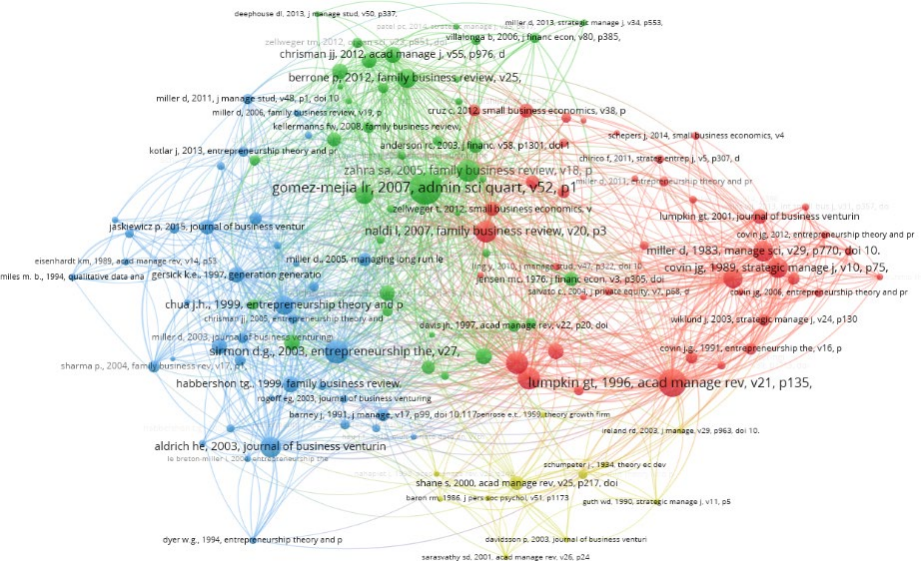
Network visualization for co-cited references

We mapped a co-citation network to visualize family firm entrepreneurship’s theoretical foundations and structure. VOSviewer showed links between items that form a co-citation link. Table 4 shows the top 20 references with the highest co-citations. In the outcomes of VOSviewer, each link contains strengths that represent a positive numerical value. The higher this value, the stronger the link. Total links strength for a reference shows the number of co-cited links of a document with other documents. Figure 2 shows the relationships of the nodes/clusters. Each node represents a document, and each line shows its co-citations links with other documents. The closer the two documents are, the stronger their relatedness. The node size for each document indicates its links with another document. The more links, the bigger the node.

**Table 4:** Top co-cited references

Sr.#	References*	Citations	Total links strength
1	Gómez-Mejía et al. (2007), *ASQ*	62	62
2	G. Lumpkin and G.G. Dess (1996), *AMR*	53	53
3	G.T. Lumpkin and G.G. Dess (1996), *AMR*	46	45
4	Sirmon and Hitt (2003), *ETP*	45	45
5	Kellermanns and Eddleston (2006), *ETP*	41	41
6	Naldi et al. (2007), *FBR*	41	41
7	Zahra et al. (2004), *ETP*	41	41
8	Zahra (2018), *FBR*	41	41
9	Aldrich and Cliff (2003), *JBV*	39	38
10	Miller (1983), *MS*	40	38
11	Covin and Slevin (1989), *SMJ*	38	38
12	Rauch et al. (2009), *ETP*	37	37
13	Berrone et al. (2012), *FBR*	36	36
14	Chua et al. (1999), *ETP*	35	35
15	Carney (2005), *ETP*	35	35
16	Chrisman and Patel (2012), *AMJ*	34	34
17	Habbershon and Williams (1999), *FBR*	30	30
18	Schulze et al. (2001), *OS*	30	30
19	Podsakoff et al. (2003), *JAP*	29	29
20	Gomez-Mejia et al. (2011), *AMA*	28	28

*ASQ = Administrative Science Quarterly; AMR = Academy of Management Review; ETP = Entrepreneurship Theory and Practice; FBR = Family Business Review; JBV = Journal of Business Venturing; MS = Management Sciences; SMJ = Strategic Management Journal; AMJ = Academy of Management Journal; OS = Organization Science; JAP = Journal of Applied Psychology; AMA = Academy of Management Annals

To reduce the complexity of the visualization, we only integrated those references that were often cited and can thus be considered particularly representative of the field. Following Lampe et al. (2020), we used a threshold of 2% (570 × 0.02 = 11) for co-cited references, indicating that references should be cited 11 times by the overall database of 570 articles. Of all 35,022 cited references, 189 were cited at least 11 times, from which 132 were connected. The top publications in terms of maximum co-citation links were Gómez-Mejía et al. (2007), G.T. Lumpkin and G.G. Dess (1996), Sirmon and Hitt (2003), Lumpkin and Dess (2001), and Zahra (2005).

We found four different clusters (red, green, blue, and yellow), as shown in Fig. 2. We read the papers in each cluster to understand the main intellectual foundations explaining family firm entrepreneurship, focusing on highly co-cited references in each cluster with strong links. Each cluster displayed several sub-topics. Consequently, we clustered the co-cited documents into more coherent sub-fields that distinguish different yet related intellectual bases of the research on family firms’ entrepreneurship, and explored four theoretical approaches (shown by color): Cluster one focuses on SEW (red with 41 documents), cluster two captures entrepreneurial orientation (green, also with 41 documents), cluster three comprises family embedded resources (blue with 39 documents), and cluster four agency theory (yellow with 11 documents).

#### SEW (red)

Literature in this cluster is centered around the theoretical idea that family firms at least partially make decisions based on non-financial considerations grounded in preserving SEW. In this cluster, researchers pay attention to three sub-research areas: the *behavioral factors*, *decision/strategy*, and *generational approaches* connected to SEW. Berrone et al. (2012) for instance say that SEW is the most vital differentiator in family firms. They further discuss the operationalization of each dimension of SEW and their link with other theoretical slants. Chrisman and Patel (2012) shed light on long-term and short-term family and economic goals that cause variation in behaviors (e.g. investment decisions). Gomez-Mejia et al. (2011) demonstrate that contingency factors (e.g. firm size, firm hazard, family stage, and the presence of non-family shareholders) influence SEW when managers make decisions to enhance performance. Kellermanns and Eddleston (2006) discuss the role of generation involvement and technological opportunities in family firm entrepreneurship, while Habbershon and Pistrui (2002) shed light on the family-influenced ownership groups in the persuasion of transgenerational wealth during entrepreneurial decision-making. Overall, studies in this cluster demonstrate that entrepreneurship in family firms essentially encircles SEW. While making entrepreneurial decisions, we argue that family firms maintain a high cognition of their SEW, which can affect their actions as a result.

#### Entrepreneurial orientation (green)

Documents in this cluster emphasize the main roots of entrepreneurial activities by emphasizing three major research areas: *entrepreneurial behaviors*, *strategic behaviors*, and *firm performance*. Covin, Miller, and Zara are the main authors in this cluster, shedding light on entrepreneurial behaviors and performance. Considering the most weighted articles, four studies (Covin and Slevin 1989, 1991; Kellermanns and Eddleston 2006; Miller 1983) discuss the relationship between the entrepreneurial and strategic behaviors of firms. However, another group of scholars (G.T. Lumpkin & G.G. Dess, 1996; Rauch et al., 2009; Wiklund & Shepherd, 2003) discuss the relationship between entrepreneurial orientation and firm performance. Other main authors in this cluster, for instance, Covin and Slevin (1991), outline a conceptual model of entrepreneurship by describing entrepreneurship as a strategic posture represented by a firm’s risk-taking behaviors, competitive aggressiveness, proactiveness, and innovativeness. Miller (1983) discovered the determinants of entrepreneurship in various firms (simple, planning, and organic), which are characteristics of leaders in simple firms, product-market strategies in planning firms, and environment and structure in organic firms. Through the meta-analysis of 51 studies, Rauch et al. (2009) identify several mechanisms that moderate the connection between entrepreneurial orientation and firm performance. Overall, the literature in this cluster reveals that entrepreneurial orientation is another major theoretical approach in family firms’ entrepreneurship.

#### Family embedded resources (blue)

This area of research discusses the resources (tangible and intangible) and the resource-based view (RBV) in relation to value creation and firm performance. Most of the studies in this cluster specifically discuss the interaction of two research topics: *resources* and *value creation* in family businesses. In particular, Aldrich and Cliff (2003), Habbershon et al. (2003), Sirmon and Hitt (2003), and Sirmon et al. (2008) discuss the phenomenon of how firms manage their resources to maximize their values and gain advantages. Aldrich and Cliff (2003) argue that transitions, resources, norms, and attitudes influence new venture creation and outcomes in family firms. Habbershon et al. (2003) develop a unified system model of wealth-creating performance of family firms that integrates capabilities and resources (generated by families’ systems) with their potential for transgenerational wealth creation. Similarly, Habbershon and Williams (1999) propose a theoretical base for “familiness” (distinctive resources of firm results from family involvement) for assessing competitive advantage and distinctive performance capabilities. Cabrera-Suárez et al. (2001) use two theoretical approaches, the RBV and knowledge-based view, to understand the nature and transfer of knowledge within family firms for developing competitive advantage. To summarize, studies in this cluster use various resources (tangible and intangible resources) and competences that are based on the theoretical foundation of the RBV in the field of family firm entrepreneurship.

#### Agency theory (yellow)

This research area mainly discusses *agency cost* and *stewardship* in family businesses. For instance, Corbetta and Salvato (2004) suggest stewardship theory to counterpart the agency framework in enlightening entrepreneurial and organizational behaviors. They further suggest that the variances in organizational performance are not merely linked to family involvement, but to the pervasiveness of agency or stewardship relationships as well. The study by Jensen and Meckling (1976) is popular in family business literature because it integrates elements from the three theories of agency, property rights, and finance to develop a theory of the firm’s ownership structure. Schulze et al. (2001) contribute to agency theory through the lens of family firms by extending previous agency models (Jensen and Meckling’s). They shed light on how family dynamics, i.e. altruism, intensify agency problems experienced by owners of privately held firms.

Villalonga and Amit (2006) describe family ownership as valuable when the founder is the CEO or chairperson. However, dual shares, voting agreements, and pyramids mitigate founders’ premiums. Descendants as CEOs and their conflict between family and non-family shareholders destroy firms’ values in family firms. Davis et al. (1997) propose a model to reconcile the difference between governance and subordinates (e.g. agency theory and stewardship) by considering several psychological and sociological characteristics that are antecedents to the principal-steward relationship. Based on the documents in this cluster, we conclude that entrepreneurship in family firms is significantly influenced by family ownership structure. Consequently, we deem agency theory to be a theoretical approach that plays a major role in entrepreneurial behaviors and entrepreneurial decision making in family firms.

### Bibliographic coupling

We conducted a bibliographic coupling analysis to identify the recent developments in the field (i.e. research clusters of papers that have yet to be extensively cited).

There are different ways to select core sets of articles for a bibliographic coupling analysis. For instance, some authors suggest focusing on time periods (e.g. seven years) (Bernatović et al. 2021) or the last two years in the data set (Galvagno and Pisano 2021), while others recommend minimum citations of a document such as five (Baier-Fuentes et al. 2019) or 10 citations per document (Niebla-Zatarain et al. 2020). We followed the second recommendation, using a minimum of seven citations per document, approximately one-third of the average citations of the database (12,046/570 = 21.13 and divided by 3).

**Fig. 3:**
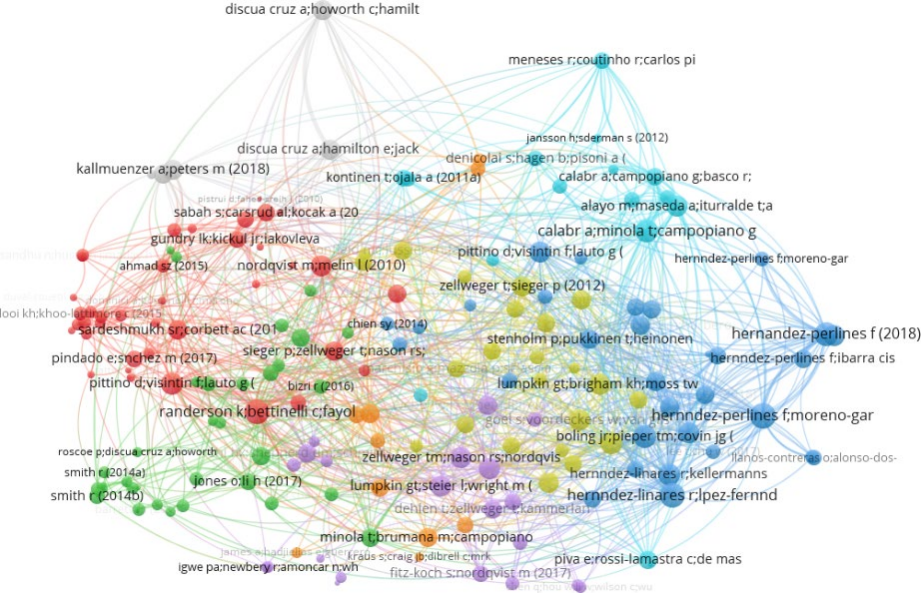
Network visualization for coupling documents

Out of 570 documents, 300 met the condition of a minimum of seven citations per document. However, only 185 of these were coupled (of which 98 were highly coupled, as shown in Table 5). Out of 185, the top 20 coupled documents with the highest total link strength are shown in Table 6. Figure 3 illustrates the network visualization for bibliographic coupling documents. The three documents with the highest number of total couplings are Lundmark et al. (2019), Nordqvist et al. (2013), and Hernández-Perlines et al. (2019). Unsurprisingly, the articles by Nordqvist and Hernández-Perlines can be found in the 20 top-cited authors (see Table 3). Moreover, Nordqvist was also listed as one of the most productive authors (10 documents), as well as in the total links strength category. The article by Nordqvist, Wennberg and Hellerstedt (2013) reviews previous literature from 1974 to 2010 on family firms’ succession through the lens of entrepreneurial process. Because of the significant role of the research area (entrepreneurship in family firms), this article has been repeatedly cited.

**Table 5:** Description of Highly Coupled Documents

Characteristic	Number of Studies
Data	Survey 31 Secondary data 10 Survey and secondary data 7 Interviews 21 Case study 6 Mixed method 23
Size	Large firms/Companies 48 SMEs 23 SMEs and large firms 2 New ventures, startup intention 25
Industry	Cross-industry 44 Hospitability and tourism 6 Manufacturing 3
Country	Europe 72 Asia/China 15 Other 11
Focus	Comparison of family-owned and non-family firms 19 Only family firms 79

**Table 6:** Top 20 coupled documents

Documents*	Citations	Total links strength
1. Lundmark et al. (2019), *ETP*	7	133.29
2. Nordqvist et al. (2013), *SBE*	70	132.73
3. Hernández-Perlines et al. (2019), *IEMJ*	11	125.58
4. Madison et al. (2016), *FBR*	85	120.96
5. Randerson et al. (2015), *JFBS*	74	117.71
6. Hernández-Linares and López-Fernández (2018), *FBR*	21	116.89
7. Stewart and Hitt (2012), *FBR*	232	113.96
8. Pittino, Martínez, et al. (2018), *JBR*	31	111.00
9. Hernández-Perlines and Ibarra Cisneros (2018), *Sustainability*	21	108.75
10. Stanley et al. (2019), *FBR*	18	104.93
11. Calabro et al. (2016), *EJIM*	16	99.00
12. Bettinelli et al. (2017), *JSBM*	19	98.00
13. Boling et al. (2016), *ETP*	89	97.00
14. Kraiczy et al. (2014), *JBR*	53	91.71
15. Pittino, Visintin, et al. (2018), *FBR*	10	91.00
16. Deb and Wiklund (2017), *JSBM*	19	90.66
17. Rau et al. (2019), *JFBS*	14	89.00
18. Tasavori et al. (2018), *ISBJ*	12	87.99
19. Sciascia et al. (2012), *SBE*	145	87.98
20. Cherchem (2017), *JFBS*	23	87.00

*JFBS = Journal of Family Business Strategy; ETP = Entrepreneurship Theory and Practice; FBR = Family Business Review; JBV = Journal of Business Venturing; MS = Management Sciences; SBE = Small Business Economics; IEMJ = International Entrepreneurship and Management Journal; EJIM = European Journal of International Management; JSBM = Journal of Small Business Management; ISBJ = International Small Business Journal.

We analyzed 185 documents in coupling clusters, of which 98 (see Table 5) are highly influential documents (high citations and highly coupled), most of which are considered in coupling clusters based on their sub-topics. Of these, 31 documents use a questionnaire/survey method, 21 are qualitative interview-based, seven documents are based on both survey and secondary data, six are case study based, while the rest rely on mixed methods (observations, reports, and other sources). Most documents emphasize family firm entrepreneurship in large firms and companies, 23 papers were conducted in SMEs, and only two papers were carried out in SMEs and large firms. In comparison, 25 documents focus on new ventures, startups, and entrepreneurial intentions. The majority of the studies focus on family firm entrepreneurship in cross-industries. Six articles furthermore focus on hospitality and tourism, while only three were conducted in manufacturing firms.

Most studies focus on entrepreneurship in Europe (72), followed by Asia (15), while the rest of the documents (11) were conducted in other countries, including the UK and Australia. Moreover, (19) articles investigate entrepreneurship compared to family and non-family-owned firms, while 79 solely focus on family firms.

The additional analysis revealed seven current research streams on entrepreneurship and family business: entrepreneurial motivation (red cluster: 43 documents), gender and success (green cluster: 35 documents), entrepreneurial orientation (blue cluster: 31 documents), individual and firm-level characteristics (yellow cluster: 24 documents), family embedded network (purple cluster: 21 documents), family firms’ internationalization (sky blue cluster: 17 documents), and family heterogeneity (orange cluster: 14 documents).

#### Entrepreneurial motivation (red)

Studies in this cluster examine the *social and economic motivations* of individuals and firms to *start/perceive new ventures*. For instance, Dominici et al. (2019) investigate non-economic motives (attitudes, lifestyles, and passion) among owners who manage small family-owned enterprises. Ramadani et al. (2017) research how individuals are motivated towards success in the family bookkeeping business. Peters and Kallmuenzer (2018) focus on entrepreneurial motives (financial and non-financial) perceived by owners/managers that influence the family business’ performance. Hanson et al. (2019) study how relational processes shape entrepreneurial culture and resilience across generations in family businesses. Liguori et al. (2018) scrutinize the role of self-efficacy, gender, minority status, and environmental/background inputs (prior experience of work, entrepreneurship, and family business) in entrepreneurial intention. Pittino, Visintin, et al. (2018) examine family embeddedness situations and the goals as well as qualities of individuals with a family business background in entrepreneurship and underpinning a new family venture. Most of the studies in this cluster shed light on individuals’ interests, personal motivation, and future goals through the lens of entrepreneurial intention. Literature in this cluster in particular discusses the factors that foster or hinder the entrepreneurial motivation of family members, managers, CEOs and their siblings etc. in the context of family firms.

#### Gender and succession (green)

Articles published in this cluster discuss the interaction and *role of gender*, *succession*, *social capital*, and *generation* through the lens of entrepreneurial activities in the family business. Bizri (2016) for instance reveal that social capital significantly influences succession that triggers entrepreneurial behaviors in family businesses. Bizri (2017) study characteristics of refugees’ entrepreneurial startups in family firms through the lens of the social capital theory, finding that the five characteristics of a “one-way-ahead” attitude, a pseudo-family business perception, collective bootstrapping, a distinctive network structure, and opportunity-seizing proliferation boost entrepreneurial opportunities in their host countries. Fernandes and Mota-Ribeiro (2017) examine gender discourses and their influence on starting new businesses in family firms. Aljuwaiber (2020) sheds light on gender, age, and behaviors as they relate to entrepreneurial perceptions. Gherardi and Perrotta (2016) study how daughters assume gender discrimination in succession practices when approaching entrepreneurship in family firms. Using an intersectionality framework while taking gender and social class into consideration, Constantinidis et al. (2018) discuss the family’s role in women’s entrepreneurial success in the socio-cultural environment. Overall, studies in this cluster emphasize gender roles and succession processes that influence entrepreneurial activity in family firms. We see that gender (son and daughter, male CEO vs. female CEO) and the succession process exercise different influences on the entrepreneurial process due to prevailing inequality within their social relationships and authority.

#### Entrepreneurial orientation (blue)

Scholars in this cluster focus on connecting entrepreneurial orientation with other sub-factors, i.e. *SEW*, *environmental factors*, and *firm performance*. For instance, Alonso-Dos-Santos and Llanos-Contreras (2019) discuss how SEW and entrepreneurial orientation influence the performance of family firms during natural disasters. Hernández-Perlines et al. (2021) reveal that concern for SEW positively moderates the influence of entrepreneurial orientation on family firm performance. Hernández-Perlines et al. (2019) find that SEW contributes to a higher entrepreneurial orientation in family businesses. Zachary et al. (2017) focus on how environmental changes influence entrepreneurial activities in the family business, as well as and how favorable environments encourage new generations toward environmental activities in family businesses (Casillas et al. 2011; Llanos-Contreras et al. 2020) scrutinize how the interaction of SEW and entrepreneurial orientation influences family firms’ risk-taking behaviors in a post-disaster scenario. Hughes et al. (2018) discuss the role of entrepreneurial orientation, exploration and exploitation activities in the performance of family firms. This current research stream demonstrates how entrepreneurial orientation influences family firm performance when they strongly adhere to SEW and under different environments. Entrepreneurial orientation is clearly an important theoretical foundation and current stream of research in the field of family firm entrepreneurship.

#### Individual-family characteristics (yellow)

The research papers published in this cluster examine the role of *managerial and family characteristics* regarding the *entrepreneurial outcomes* of *family firm performance*. Most of the studies in this cluster are empirical, emphasizing the moderating role of managerial and firm-level demographic factors between firms’ resources and capabilities, as well as entrepreneurial outcomes in family and non-family-owned firms. For instance, Block et al. (2016) use empirical data from 12,000 individuals from 40 countries to reveal that socio-demographic, occupational, and entrepreneurship-related factors influence individuals’ behaviors and incentives to work with family firms. Goel et al. (2013) reveal that the existence of one or more external directors could have a direct and moderating effect on the relationship between CEOs’ empathy and SEW. Marchisio et al. (2010) study the interaction of corporate venturing with individuals and firm-level factors in family firms, demonstrating that corporate venturing can have mixed influences (positive, negative, or possibly both) at the family and individual levels in the presence of moderating factors. Cherchem (2017) addresses the moderating role of generation involvement between entrepreneurial orientation family firm performances. Overall, most of the studies in this cluster use a quantitative approach (primary and secondary data) to assess how family and non-family owned firms can sustain their entrepreneurial performance through various factors (managerial and firm level) and resources. This provides hints for methodological advancement and statistical analyzes in the field of family firm entrepreneurship.

#### Family embedded network (purple)

Studies in this cluster discuss the interaction of *capabilities*, *social networks* (social capital), and *entrepreneurial processes* in family businesses. Shi et al. (2015) emphasize the relationship between trust, social capital, and the entrepreneurial process among family businesses, suggesting that managers should emphasize social networks before assessing resources to engage in the entrepreneurial process. Shi and Dana (2013) stress owners’/managers’ socialization (social network outside the business) as an important capability between market orientation and the entrepreneurial process in Chinese family firms. Daspit and Long (2014) extend previous models of resource accumulations (Khayesi et al. 2014) by including the structural and relational dimension of an entrepreneur’s social capital network to elaborate more on entrepreneurial kinship network types in family firms.

Moreover, Mickiewicz and Rebmann (2020) examine how trust (relationship with external bodies) enables entrepreneurship in family firms in uncertain situations, and how advanced technological capabilities help acquire useful information to increase trust. Kotlar and Sieger (2019) shed light on the conditions under which family managers and non-family managers support entrepreneurial activities. Chen et al. (2016) reveal that adapting international accounting standards improves access to international funds in mature entrepreneurial family firms. This stream of research describes entrepreneurship in family businesses under social capital, revealing to what extent family business networks with outside partners (financial institutions, foreign businesses, political bodies, suppliers, etc.) affect their entrepreneurial activities. Indeed, networking ties and external relationships encourage individuals to start new businesses and engage in entrepreneurship (Klyver and Arenius 2022).

#### Family firms’ internationalization (sky blue)

The articles in this cluster discuss *international entrepreneurship*, *international opportunity recognition*, and the *internationalization* of family businesses. Ratten et al. (2017) demonstrate that entrepreneurial approaches (innovative and risk-taking) are crucial for the internationalization of family firms. Alayo et al. (2019) shed light on the importance of family involvement, while Calabrò et al. (2017) scrutinize the influence of family governance on internationalization and internationalization opportunity recognition in the family business. Moreover, Denicolai et al. (2015) examine the influence of entrepreneur demographic factors (experience and education etc.), while Singh and Kota (2017) state that family firms are entrepreneurial in nature, and younger family firms take advantage of innovation for international market entry. Chen et al. (2015) test the role of demographic factors of CEOs in international expansion strategy in family-owned SMEs. Studies in this cluster discuss various factors that influence international opportunity recognition, international entrepreneurship, and the internationalization success of family businesses. Changing environments in the current era are proof of more international entrepreneurship opportunities for family businesses (Zahra 2022).

#### Family heterogeneity (orange)

Researchers in this cluster address the *heterogeneous factors* that influence the *entrepreneurial process* in family businesses. For instance, Adjei et al. (2019) examine how the children, spouses, and siblings of entrepreneurs with different skills influence family business performance. Bird and Zellweger (2018) test the role of the entrepreneurial team (especially siblings and spouses of entrepreneurs) and industry as they relate to experiencing heterogeneity when generating family firm growth. Coad and Timmermans (2014) also examine how heterogeneous and diverse teams influence the growth of new family ventures. Minola et al. (2016) assess the impact of enterprising family dynamics (such as the birth of a child or children leaving home) on the entrepreneurial activities and motivation of entrepreneurial venturing into family businesses. Schjoedt et al. (2013) check the influence of entrepreneurial team formation and composition on new venture creation in family firms. Moreover, Adjei et al. (2019) discuss the importance of entrepreneurs’ children and spousal relationships on family firm performance. James et al. (2020) address various heterogeneity approaches (e.g. generation, culture, social actors, etc.) in entrepreneurial families. Family businesses are heterogeneous in nature (generation, ownership, governance structure, etc.), causing a significant variation in entrepreneurial outcomes. Depending on the situation, heterogeneity can foster or hinder entrepreneurship in family businesses, helping explain researchers’ strong interest in studying heterogeneity in family firm entrepreneurship.

### Linking co-citation and coupling clusters

We further analyzed which co-citation clusters were cited by the coupling clusters to recognize the theoretical roots of each bibliographic coupling cluster in family firms’ entrepreneurship. This allowed us to identify which theoretical paradigms primarily influence the thinking in each current discourse, and where potential white spaces for future research can be identified.

**Fig. 4:**
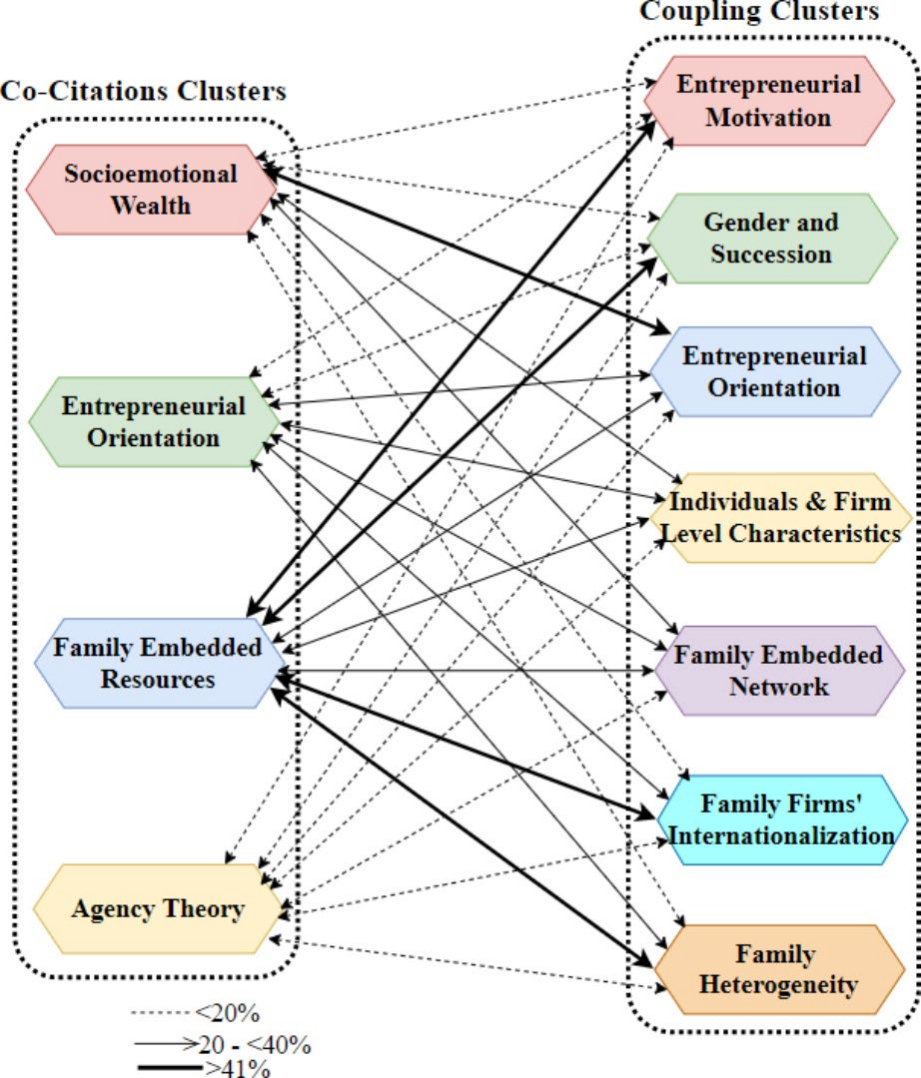
Relationship among co-citation clusters and coupling clusters

Table 7 illustrates the number of papers published in each co-cited cluster cited by each coupling cluster. For example, the coupling cluster cites 16 papers of the SEW co-citation cluster on entrepreneurial motivation. In other words, 20% of entrepreneurial motivation references belong to the references of the SEW cluster. Figure 4 maps the cross-checking references of co-cited and coupling clusters. The bold solid lines indicate 40% or more citations, the thin solid lines show 20–40%, while the dotted lines illustrate less than 20% citations of the specific co-cited cluster by the particular coupling cluster. The thin and dotted lines indicate a lack of research on the relationship between the particular topics or research areas, providing potential impulses for future studies.

**Table 7:** Co**-**cited reference cross**-**checking coupling clusters

Coupling Clusters	Co-Citations Clusters			
	SEW	EO	Family Embedded Resources	Agency Theory
Entrepreneurial motivation	16 (20%)	11 (13%)	42 (51%)	13 (16%)
Gender and succession	5 (5.6%)	10 (11.2%)	55 (61.8%)	19 (21.3%)
Entrepreneurial orientation	90 (47%)	47(24.5%)	50(26%)	5(2.5%)
Individuals’ firm-level characteristics	45(33.3%)	34(25.2%)	53(39.3%)	3(2.2%)
Family embedded network	27(22.9%)	42(35.6%)	41(34.7%)	8(6.8%)
Family firms’ internationalization	12(20%)	20(33.3)	24(40%)	4(6.7%)
Family heterogeneity	5(10.6%)	14(29.8%)	25(53.2%)	3(6.4%)

Note: the numerical values indicate the number of cited articles in each co-citations cluster by each coupling cluster. For instance, 90 articles published in SEW (co-citation cluster) are cited by EO (coupling cluster)

Figure 4 shows that the papers published in the co-cited clusters SEW, entrepreneurial orientation, and family embedded resources are highly cited by the coupled clusters of entrepreneurial orientation, individual and firm-level characteristics, and family embedded networks. It demonstrates that the research streams of entrepreneurial orientation, individual and firm-level factors, and family embedded networks are highly dependent on SEW, entrepreneurial orientation, and family embedded resources. For instance, considering a few mostly coupled documents from the entrepreneurial orientation cluster, Hernández-Perlines et al. (2019) discuss the interaction of SEW and entrepreneurial orientation in family businesses. Schepers et al. (2014) focus on the moderating role of SEW between entrepreneurial orientation and the performance of family firms. Hernández-Linares et al. (2018) shed light on the relationship between market and learning orientation and entrepreneurial orientation through the lens of the RBV. Articles published in the cluster of individual family level characteristics are rooted in SEW, entrepreneurial orientation, and the RBV. For instance, Goel et al. (2013) examine the influence of CEO empathy on SEW in family firms. Martínez et al. (2016) describe how family influences knowledge transfer to entrepreneurial orientation in family firms. Bacq and Lumpkin (2014) discuss how social entrepreneurs could learn from family entrepreneurship to address the challenges of achieving competitive advantages, bringing into line multiple stakeholders, and endorsing sustainable solutions through the lens of the RBV and stewardship theories.

Moreover, articles published in the family embedded network are theoretically connected with SEW, entrepreneurial orientation, and the RBV. For instance, Daspit and Long (2014) demonstrate how social and entrepreneurial networks/capital influence moral hazards in family firms. Le Breton-Miller et al. (2015) discuss under which conditions of agency theory, behavioral agency perspectives, and the RBV family firms are more or less entrepreneurial. Shi et al. (2015) emphasize the relationship between trust, social capital, and entrepreneurial processes among family businesses. Igwe et al. (2018) determine the role of artisanal capability/skill in entrepreneurial decision-making among family enterprises in situations where individuals have connected with training and non-training institutions.

Specifically compared to other roots, family embedded resources (e.g., the RBV) are the basic root of coupling clusters: entrepreneurial motivation, gender and succession, entrepreneurial orientation, individuals and firm-level characteristics, family embedded networks, family firm internationalization, and family heterogeneity. For example, while arguing the RBV, Pindado and Sánchez (2017) examine the influence of entrepreneurs’ capabilities and resources on the entrepreneurial process in family firms. Tolentino et al. (2014) discuss the role of various resources that foster the entrepreneurial intention of individuals with family business experience and backgrounds.

However, the rest of the clusters are marginally connected, indicating that the theoretical foundation, namely agency theory, has been paid little attention in the current debate on family firm entrepreneurship. Also, SEW, entrepreneurial orientation, and agency theory have rarely been discussed in current research on family firm entrepreneurship such as entrepreneurial motivation, family firm internationalization, gender and succession, and the family embedded network. Overall, it is evident from the integrated figure that family business scholars today should focus on SEW and agency theory as a theoretical foundation for researching the importance and role of entrepreneurial motivation, family firm internationalization, gender and succession, and family embedded networks in family firm entrepreneurship. We discuss these gaps in detail in the future research directions in Table 8.

**Table 8:** Potential Areas for future research

Research topics	Gaps	Possible research questions	Possible theories and approaches
1. SEW and the internationalization of family firms	• The role of the dimensions of SEW in international entrepreneurship and international opportunity recognition	• Do the dimensions of SEW influence international entrepreneurship opportunity? • Does international opportunity recognition mediate or moderate between SEW internationalization?	• SEW theory • Internationalization theory
2. SEW and entrepreneurial motivation	• SEW and individual and firm-level entrepreneurial motivation in family and non-family firms • The interaction of SEW, generational, and entrepreneurial motivation	• Does SEW influence individual and firm-level entrepreneurial motivation in family firms? • Does generation play a role between SEW and entrepreneurial motivation?	• SEW theory • Upper echelon theory
3. Entrepreneurial orientation and agency costs in family businesses	• The influence of agency conflicts on entrepreneurial activities in family firms • Agency conflicts and entrepreneurship in family and non-family firms	• How do agency conflicts affect entrepreneurial activities in family firms? • How do agency problems influence the entrepreneurial process in family and non-family firms?	• Agency theory
4. SEW and gender and succession	• The casual interactions of the dimensions of SEW, gender and succession	• Does gender or succession process moderate between SEW dimensions and entrepreneurship in family firms? • Do SEW dimensions play any mediating role between gender, succession and family entrepreneurial processes?	• SEW theory
5. SEW and family heterogeneity	• Heterogeneity and SEW and their role in the entrepreneurial process of family firms • The moderating role of gender and succession between SEW, heterogeneity and entrepreneurship	• How do different types of heterogeneity (e.g. generational, control, and governance) affect SEW and entrepreneurial activities? • Under which circumstance does heterogeneity promote entrepreneurship in family firms?	• SEW theory
6. Entrepreneurial orientation and gender and succession	• Examining the relations between gender, succession and entrepreneurial processes • Systematic review and meta-analysis of this relationship	• How do dimensions of entrepreneurial orientation, gender and succession process influence each other? • How do these factors play moderating or mediating roles in family entrepreneurship?	• Entrepreneurship theory
7. Agency conflict, family heterogeneity, individual and firm-level characteristics	• The nexus between agency conflicts, heterogeneity, individual and firm-level characteristics and entrepreneurship	• How do agency conflicts affect the association between individuals and firm-level characters and entrepreneurship? • How do agency conflicts affect the influence of family heterogeneity on entrepreneurial activities?	• Agency theory • Upper echelon theory
8. Internationalization of family firms through the lens of agency theory	• Agency conflict between family and non-family members to acquire and manage resources for internationalization • Barriers and challenges of internationalization to family firms through the lens of agency theory	• How does agency conflict influence entrepreneurial activities and the management of resources in family firms? • What are the barriers and challenges to family firms entering new markets in terms of agency?	• Agency theory • Internationalization theory
9. Agency conflicts and family-embedded network	• The role of agency in entrepreneurial networks (centrality and multiplexity) • Agency conflicts between individual and firm-level networking and entrepreneurship	• How do agency conflicts affect entrepreneurial networks in family and non-family firms? • Do agency conflicts affect the relationship between family heterogeneity and entrepreneurial networks?	• Agency theory • Social network theory

## Discussion and conclusions

Research on family firms’ entrepreneurship has been rapidly growing, most notably in light of the increasing number of family businesses (Gagne et al. 2021) and their contributions to GDP and employment (Alayo et al. 2021). The literature here has focused on various aspects, including the strategies, benefits, barriers, and enablers of family firms’ entrepreneurship (e.g. Aljuwaiber, 2020; Bacq & Lumpkin, 2014; Boers & Henschel, 2021; Feldmann et al., 2020; Peters & Kallmuenzer, 2018). However, the vast amount of studies across various niche areas makes it difficult to categorize the intellectual structure of family firms’ entrepreneurship. There is surprisingly no recent bibliometric study on family firm entrepreneurship. Although a few qualitative studies in the field have shed light on main research streams that are limited to narrow fields (Cardella et al. 2020; Minola et al. 2021; Randerson et al. 2015), it remains challenging to summarize, recognize, and address theoretical foundations and current streams in a qualitative review. To fill this gap, this updated bibliometric study on family firm entrepreneurship maps the family firms’ domain, sheds light on its respective intellectual structure, and recognizes the emerging research streams in the field.

### Implications and an agenda for future research

Our research provides a unique recipe for future scholars eager to study family firms’ entrepreneurship. We have proposed several directions for future researchers to articulate the research stream in a better way. High citation amounts of the current streams indicate that the demand for researching family firms’ entrepreneurship is further increasing, perhaps with the potential to generate a diversity of research areas.

Our analysis reveals the following realms that could/should be of interest for future research.

1. *SEW and the family firms’ internationalization*: Our integrated figure illustrates a poor connection between SEW and the internationalization of family firms. We in turn suggest that authors could examine the role of the dimensions of SEW in international entrepreneurship and international opportunity recognition. Authors could specifically focus on answering the question “Do the dimensions of SEW influence international entrepreneurship opportunity? Does international opportunity recognition mediate between SEW-internationalization?”
2. *SEW and entrepreneurial motivation in family firms*: The integrated model displayed a poor connection between SEW, entrepreneurial motivation and gender and succession. Although Feldmann et al. (2020) reveal that gender identity significantly influences entrepreneurship decisions in family firms, how gender identity influences the path between SEW and entrepreneurial motivation in various regions and cultures remains poorly discussed and insufficiently researched.
3. *SEW and gender and succession*: Apart from the existing literature, we suggest that future researchers test the casual interactions between the dimensions of SEW, gender and succession, and their relationship to family firm entrepreneurship. The moderating mechanisms (gender and succession) and mediating factors (SEW) can be assessed between/among the casual relationship.
4. *Entrepreneurial orientation and agency cost in family businesses*: Our cross-checking model detected a gap for examining the influence of agency conflicts on entrepreneurial activities in family and non-family businesses. There is also an opportunity for family business scholars to conduct systematic literature or meta-analysis to extract the solid connections between these factors and their role in a family business.
5. *SEW and family heterogeneity*: Family firms have significant heterogeneity in ownership and governance systems. Given this dissimilarity, it is important to determine whether the interaction of SEW and family heterogeneity influences entrepreneurial activities. Heterogeneity is a broad concept (and might also include a few dimensions of SEW) (Chua et al. 2012). In particular, gender, succession, governance and generations can be considered in the relationship between SEW and entrepreneurial activities in various family businesses (small, large, manufacturing, trading and services, etc.). It would furthermore be interesting to assess various barriers in family firms’ opportunity- and necessity-based entrepreneurship (Khanin et al. 2022), most notably regarding family firms and their social, economic, political, and environmental factors. And in the era of digitalization, while preserving SEW, it could be interesting to assess how these businesses use artificial intelligence to innovate their business models and create values (Åström et al. 2022; Rubio-Andrés et al. 2022).
6. *Entrepreneurial orientation and gender and succession*: Based on our findings, we suggest future researchers examine the relations between gender, succession, and entrepreneurial processes. Here we strongly recommend conducting systematic reviews or meta-analyzes of the aforementioned relationship to understand how these factors are related and play a role in family firm entrepreneurship.
7. *Agency cost and family heterogeneity in family businesses*: Family business scholars have extensively compared family and non-family businesses through the lens of entrepreneurship. Given the connection between entrepreneurial orientation, family heterogeneity, and agency theory, we identified a weak link. Moreover, agency cost is not limited to family firms; strategic initiatives in non-family firms are instead also significantly influenced by various agency problems (Chatzopoulou et al. 2021). It could be a better idea to assess under which relevant mechanisms (e.g., culture, religions, freedom, origins, dual nationality, and succession) the agency cost influences entrepreneurial orientation in family and non-family firms.
8. *Internationalization of family firms through the lens of the agency theory*: We have acknowledged the missing gap between agency theory and the internationalization of family firms, and know that the field can be advanced by investigating the barriers and challenges to the internationalization of family firms through the lens of these theories. Our research gap to some degree is related to the bibliometric study by Casprini et al. (2020), who discusses that family members are often not inclined towards internationalization because of the potential loss of SEW. Hence, non-family stakeholders face issues in expanding their businesses, perhaps leading to conflict in acquiring and managing resources for new market entrance. Family business scholars should emphasize how family and non-family managers adjust their resources to expand their businesses.
9. *Agency conflicts and family embedded network*s: Network ties play a key role in acquiring resources to foster entrepreneurship. However, many businesses do not have strong relationships with those outside their own four walls. Moreover, the social ties of family firms are significantly affected by the agency conflicts between agents and owners, therefore influencing their performance and risk-taking behaviors (Gomez-Mejia et al. 2001). We therefore recommend future scholars address the agency problems between various networking, social ties (with businesses, governments, and politics), and family firm entrepreneurship.

### Theoretical contributions

The contributions of this bibliometric study are threefold. First, the co-citations analysis enabled us to understand forward-looking assessment and intellectual foundations that correspond to the theoretical topics in the field of family firm entrepreneurship. The new insights into the theoretical foundations help scholars understand the progressive work in the field. For instance, we extracted major theoretical foundations that provide clear direction to scholars who are engaged in family firm entrepreneurship research. The insights of co-citations analysis facilitate researchers in building an appropriate conceptual and theoretical framework in family firm entrepreneurship studies.

Second, the bibliographic coupling reveals the current state and trends in family firm entrepreneurship research, helping researchers systemize and better understand where this field is developing. The coupling analysis shows that the current areas of family firms’ entrepreneurship have apparently changed from the past. Scholars are now engaged in a diverse field of studies while emphasizing new research streams. The clusters identified by coupling analysis in our study deliver a clear road map to future scholars, hinting that, rather than engaging in elusive areas, specific topics in the field of family firm entrepreneurship require focused attention.

Third, as we created an integrated network between the intellectual foundations and the current research trends, we were able to systematically identify missing research areas in the field of family firm entrepreneurship. This enabled us to recognize missing research areas in the field as we extracted several recommendations for future researchers. The co-citation and coupling show that entrepreneurship in family firms is not limited to one domain, but covers different contexts as well. Most topics or clusters are cited extensively and repeatedly over time. The insights emerging from this are also useful for advancing our knowledge of emerging future research topics and fields.

### Limitations of the study

Despite its contributions, our research has some limitations that should be addressed in future studies. First, we analyzed only peer-reviewed research articles in our study. Although this is commonly considered a quality indicator, future research may consider conference proceedings, and book reviews and chapters. The second limitation of this study is that we performed only co-citations and coupling analysis in VOSviewer. Scholars could dive deeper into co-author relationships and networks through co-occurrence and co-authorship analyzes to obtain greater, more nuanced information. In addition, scholars could consider other software such as UCINet to better understand the network structures based on different centrality measures (Donthu et al. 2021) that are unavailable in VOSViewer.

### Conclusion

The burgeoning literature on entrepreneurship in family business provided us the opportunity to conduct a bibliometric study which we executed to understand past, present, and future research areas in the field. We identified 570 documents that were published in Scopus and WOS during 2010–2021, and employed VOSviewer for analysis. The performance analysis shows a significant increase in the publications during the last four years. We revealed that the *Journal of Family Business Management*, *Entrepreneurship Theory and Practice*, and the *Journal of Family Business Strategy* are the most popular journals publishing in the field. Matthias Nordqvist, Franz W. Kellermanns, and Thomas Zellweger are the most productive authors, and the USA, UK, and Spain are the most productive countries doing research.

In the co-citation analysis based on the co-cited 189 references, we identified four intellectual foundations of entrepreneurship in family firms: socioemotional wealth (SEW), entrepreneurial orientation, family embedded resources, and agency theory. Furthermore, we analyzed 185 documents in coupling clusters published in different sectors, identifying seven streams of current research: entrepreneurial motivation, gender and success, entrepreneurial orientation, individual and firm-level characteristics, family embedded networks, family firm internationalization, and family heterogeneity. We then identified how the different intellectual foundations influence current research, seeing that it primarily refers to SEW and the theoretical perspective of family-embedded resources. Entrepreneurial orientation is less dominant in current thinking, and agency theory has only weak implications for current research. Based the interactions of these clusters (co-citation and coupling), we suggested several research areas for future scholars in the field of family firm entrepreneurship.

## Electronic supplementary material

Below is the link to the electronic supplementary material.

Supplementary Material 1

Supplementary Material 2

## Data Availability

This manuscript has no associated data. The data used in the figures and tables can be downloaded from WOS and Scopus.
