# Supplementary material for: Entrepreneurship in family firms: an updated bibliometric overview
Source: Rev Manag Sci. 2023 Mar 22:1–37. Online ahead of print. doi: 10.1007/s11846-023-00650-z (PMC10032270; doi:10.1007/s11846-023-00650-z)
Supplement: Supplementary file 1 — Supplementary Material 1 [file 11846_2023_650_MOESM1_ESM.pdf]

David Kloss • Dählmannsweg 22 • 26135 Oldenburg (Deutschland)
0163/4287351 • davekloss144@gmail.com

Oldenburg, Germany, February 13th, 2023

**Certificate of Proofreading**

Ladies and Gentlemen,

This letter is to confirm my proofreading of the text *Entrepreneurship in family firms: An updated bibliometric overview* from February 8th to 9th of this year.

You can best reach me at davekloss144@gmail.com for any further questions you may have.

All the best,

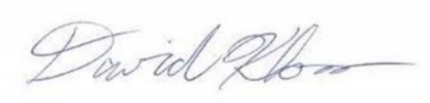

David Kloss
Dählmannsweg 22
26135 Oldenburg
Germany
