# Supplementary material for: Entrepreneurship in family firms: an updated bibliometric overview
Source: Rev Manag Sci. 2023 Mar 22:1–37. Online ahead of print. doi: 10.1007/s11846-023-00650-z (PMC10032270; doi:10.1007/s11846-023-00650-z)
Supplement: Supplementary file 2 — Supplementary Material 2 [file 11846_2023_650_MOESM2_ESM.docx]

Dear Editors-in-Chief,

Ralf Ewert, Wolfgang Kürsten, Sascha Kraus

We truly hope you and your families are fine during this special times. With this letter, we are submitting our manuscript for publication in the Review of Managerial Science‘s Special Issue on “State-of-the-Art Reviews”. With our submission we would like to ensure that this research article has not been published, accepted for publication or is under editorial review for publication in any other journal.

The paper provides a bibliometric overview by using co-citations analysis and bibliographic coupling analysis. Our study visualizes the theoretical foundations and structures of the current research on entrepreneurship in family firms.

We utilize a combination of various bibliometric analysis techniques. Using a comprehensive search query: (“entrepreneur*”) AND ("Family firm*" or "Family Business*" or "Family Enterprise*" or "Family Organization*" or "Family Organisation*" or "Family Control*" or "Family own*"), we found 1140 articles (after removing 326 duplicate items) in Scopus and WOS that have used these words in title, abstract and keywords. We manually screened each document to extract the most relevant data and dropped unnecessary documents. We then analyzed a sample of 570 documents published in Scopus and WOS during 2010-May 2021.

After an initial descriptive analysis of the research discourse based on publications and citations, we utilize VOSviewer to conduct rigorous structural co-citations and bibliographic coupling analysis at the level of individual publications. Whereas a co-citation analysis could identify the intellectual foundations of the field, bibliographic coupling helps to structure current research discourses. Finally, we linked these results with each other (i.e., showing to what extend co-cited clusters are cited by coupled clusters), to show on which theoretical foundations current research streams rely and identify white spaces to be addressed by future research.

Based on the co-citation analysis of (189 references that are cited at least eleven times out of total 35022 references), we identified four solid intellectual foundations of the research discourse: (1) socioemotional wealth (SEW), (2) entrepreneurial orientation, (3) family embedded resources, and (4) agency theory. In the visualization in Figure 1, the colors indicate to which cluster each publication belongs. The nodes show the co-cited strengths (the bigger the size of the nodes, the higher will be their co-cited links with others).

For the bibliographic coupling, we used 165 met the condition of a minimum of 20 citations per document. We found six clusters (Figure 2) in bibliographic coupling namely (1) entrepreneurial orientation, (2) family embedded network, (3) behavioral perspective and succession, (4) internationalization, (5) ownership and control, and (6) culture and narrative.

Finally, we created a network diagram to provide an integrated overview of the development of the field. Through this, we see that current research on entrepreneurial orientation significantly relies on the theoretical tenets of SEW, but significantly less on the ideas of the RBV or Agency Theory. Consequently, we could identify under researched areas that can be translated into opportunities for future research in the field of entrepreneurship and family businesses. We could identify five major research gaps:

(1) SEW, organizational culture and narrative, (2) Internationalization of family firms in the lens of agency theory and the resource base view, (3) Agency theory and start up opportunities,

(4) Entrepreneurial orientation, organizational culture and narrative and (5) Family embedded resources, ownership, culture and narrative and specific questions for future research.

Our research contributes to the current discourse on entrepreneurship in family firms in three ways: First, co-citations analysis enabled us to understand the intellectual foundations of the field. The new insights into the theoretical foundations will facilitate scholars in understanding the progressive work in the field. Second, our bibliographic coupling analysis reveals the current state and trends in research in entrepreneurship in family firms and helps researchers to systemize and better understand where this field is developing. Third, as we created an integrated network between the intellectual foundations and the current research trends, we were able to systematically identify missing research areas in the field of entrepreneurship and family firms. We extracted several white spaces to be addressed by future research.

Kind Regards,

Authors
